# Supplementary material for: Dual blockade of the lipid kinase PIP4Ks and mitotic pathways leads to cancer-selective lethality
Source: Nat Commun. 2017 Dec 19;8:2200. doi: 10.1038/s41467-017-02287-5 (PMC5736559; doi:10.1038/s41467-017-02287-5)
Supplement: Supplementary file 1 — Supplementary Information [file 41467_2017_2287_MOESM1_ESM.pdf]

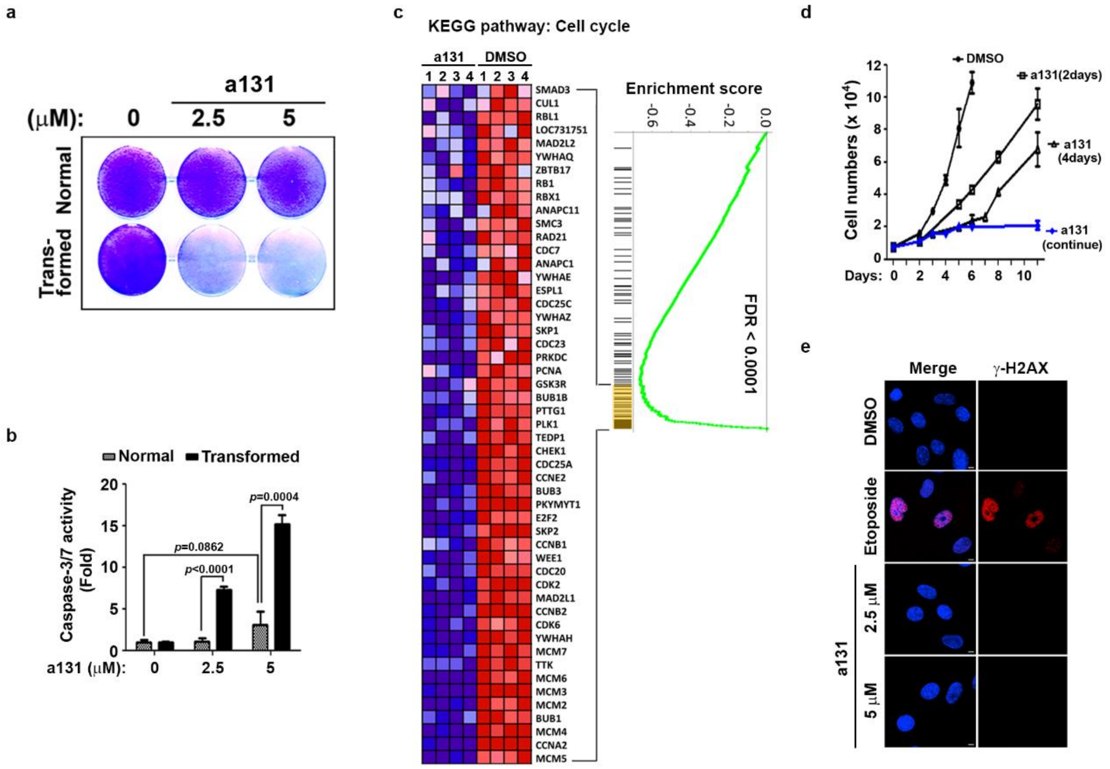

a
a131
(μM): 0 2.5 5
Normal
Trans-formed
b
Normal Transformed
Caspase-3/7 activity (Fold)
p=0.0862
p<0.0001
p=0.0004
a131 (μM): 0 2.5 5
c
KEGG pathway: Cell cycle
a131 DMSO
1 2 3 4 1 2 3 4
Enrichment score
FDR < 0.0001
d
Cell numbers (x 10^4)
DMSO
a131(2days)
a131 (4days)
a131 (continue)
Days:
e
Merge
γ-H2AX
DMSO
Etoposide
a131
2.5 μM
5 μM

Supplementary Figure 1 | Selective killing effects of a131 in transformed BJ cells without inducing genotoxic stress.

(**a**) Crystal violet assay of isogenic normal and transformed BJ cells treated with a131 for 72 h.

(**b**) Normal and transformed BJ cells were treated for 48h with the indicated concentrations of a131. Selective increase in combined activity of caspase-3/7 in transformed BJ cells by a131 treatment is shown. Mean values with ± S.D. are shown (n=3). Two-tailed unpaired t tests were performed to determine statistical significance.

(**c**) GSEA enrichment plot and heatmap of KEGG 'cell cycle' pathway genes in BJ cells treated with a131 for 24 h, compared to controls. The enrichment graph plots the enrichment scores for each gene (represented as bars), which are rank-ordered by their signal-to-noise metric between the DMSO control and a131 treated samples. Genes contributing to core enrichment of the pathway are highlighted in yellow. The per-sample expression profiles of these genes are depicted in the heatmap using an intensity-based, row-normalized color scale from blue to red, with blue indicating lower expression.

(**d**) Normal BJ cells were synchronized at the G_1 phase by serum starvation (0.1% FBS) for 2 days. Subsequently, the cells were synchronously released in fresh media with 10% FBS and then treated with 5 μM a131 for 2, 4 or 11 days. After 2 or 4 days, a131 was removed and cell proliferation continued in fresh media for up to 11 days. The total number of cells at various time points were calculated using automated cell counter (SCEPTOR, Merck) and mean values with ± S.D. are shown (n>6).

(**e**) Normal BJ cells were treated with a131 at 2.5 and 5 μM, etoposide at 100 μM or DMSO vehicle control for 48 h and subjected to immunofluorescence analysis using anti-γ-Histone H2AX (γ- H2AX) antibody and DAPI. Note that while etoposide treatment markedly induced DNA-damage visualized by staining for γ-H2AX, a131 treatment did not do so as similar to DMSO treatment. Scale bar: 5 μm.

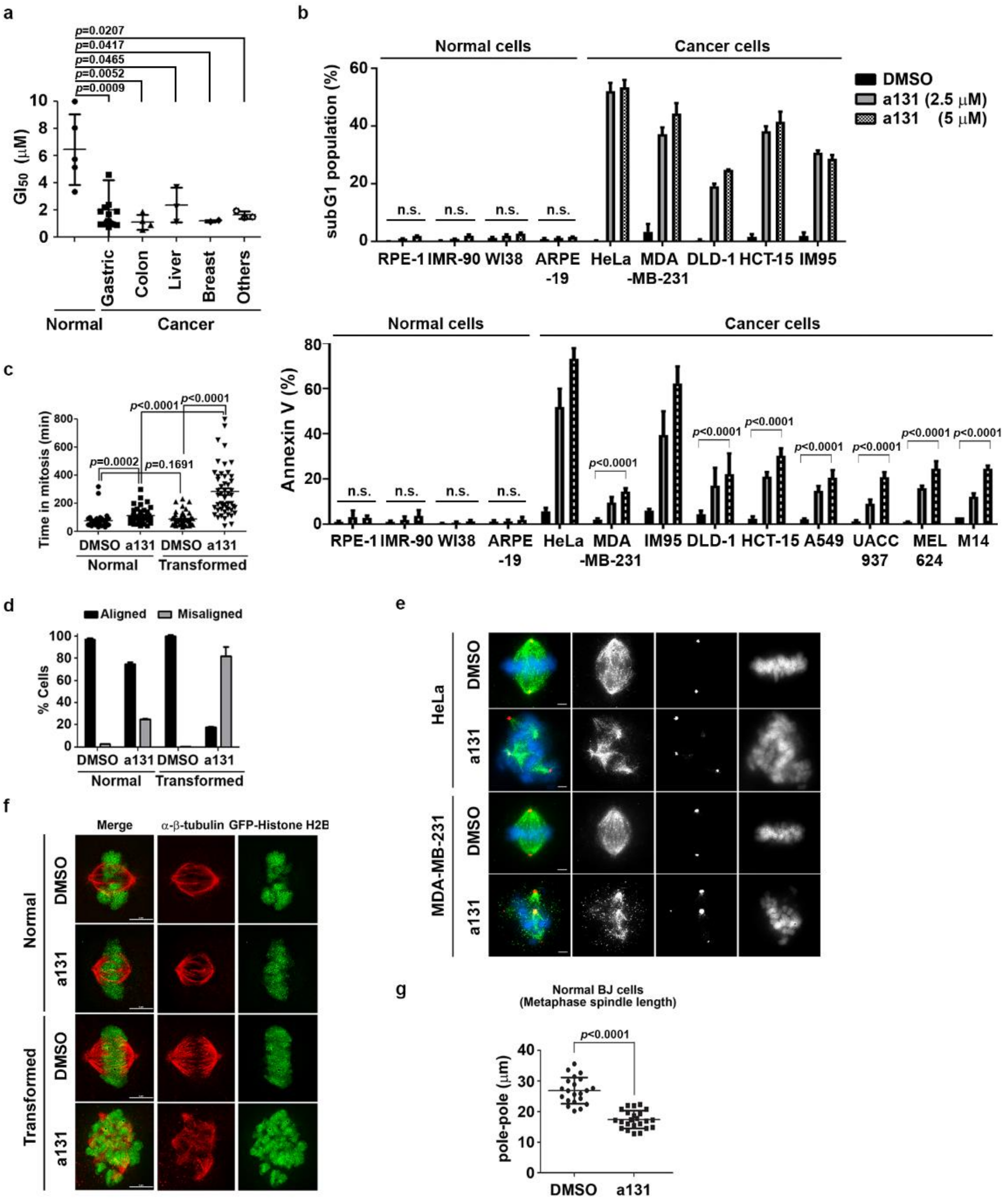
a
p=0.0207
p=0.0417
p=0.0465
p=0.0052
p=0.0009
GI50 (μM)
Gastric
Colon
Liver
Breast
Others
Normal
Cancer
b
Normal cells
Cancer cells
subG1 population (%)
DMSO
a131 (2.5 μM)
a131 (5 μM)
n.s.
RPE-1
IMR-90
WI38
ARPE -19
HeLa
MDA -MB-231
DLD-1
HCT-15
IM95
Annexin V (%)
A549
UACC 937
MEL 624
M14
p<0.0001
c
Time in mitosis (min)
p=0.0002
p=0.1691
DMSO
a131
Normal
Transformed
d
Aligned
Misaligned
% Cells
e
HeLa
MDA-MB-231
f
Merge
α-β-tubulin
GFP-Histone H2B
g
Normal BJ cells
(Metaphase spindle length)
pole-pole (μm)

Supplementary Figure 2 | Selective killing effects of a131 by inducing centrosome de-clustering in transformed BJ cells and various cancer cell lines.

(**a**) Human normal and cancer cell lines were treated with a131 at a range of different concentrations (from 0.1 μM to 40 μM) for 72 h in triplicate and cell viability was determined by MTT assay. Mean concentration values for a131 to achieve 50% growth inhibition (GI_{50}) in each groups of normal and different cancer cell lines by tissue type are plotted. Mean values with ± S.D. are shown. Two-tailed unpaired t test was performed to determine the statistical significance.

(**b**) Indicated cancer and normal cell lines were treated with a131 at 2.5 or 5 μM for 48 h. Cells were collected and stained with PI (top) or Annexin V (bottom) and subjected to FACS analysis for subG1 (<2N) population (top) and Annexin V positive population (bottom) as indication of cell death via apoptosis. Mean values with ± S.D. are shown (n>3). Two-tailed unpaired t test was performed to determine the statistical significance. n.s.: not significant

(**c, d**) Normal and transformed BJ cells stably expressing GFP-histone H2B were treated with a131 at 2.5 μM or DMSO vehicle control for 8 h and subjected to time-lapse live-cell imaging for 24 h with 5 min intervals.

(**c**) The duration of mitotic progression after nuclear envelop breakdown until completion of cell division in each randomly selected cell is presented (n>50 per condition). Mean values with ± S.D. are also shown from triplicated experiments. Two-tailed unpaired t test was performed to determine the statistical significance.

(**d**) Subsequently, cells were fixed in PFA and subjected to immunofluorescence analysis using antibodies against β-tubulin and γ-tubulin. Images were obtained using 3D-SIM super resolution microscopy and representative images are shown in Fig. 1g. Quantification of cells (n>50 per condition) with misaligned chromosomes. Mean values with ± S.D. are shown from triplicated experiments.

(**e**) Indicated cancer cell lines were treated with a131 at 2.5 μM or DMSO vehicle control for 12 h and subjected to immunofluorescence analysis as in **d** and cells were counterstained with DAPI. Images were obtained using 3D-SIM super resolution microscopy. Scale bar: 5 μm.

(**f, g**) Normal and transformed BJ cells stably expressing GFP-histone H2B were treated with a131 at 2.5 μM for 24 h. Subsequently, cells were fixed in PFA and subjected to immunofluorescence analysis using antibody against β-tubulin. Images were obtained using 3D-SIM super resolution microscopy.

(**f**) a131 treatment caused massively misaligned chromosomes with multipolar spindles in transformed BJ cells (bottom panels), but not in normal BJ cells (top panels). Scale bar: 5 μm. (**g**) Quantification of metaphase spindle length (pole-pole distance) (n>20 cells) in normal BJ cells. Mean values with ± S.D. are shown from triplicated experiments. Two-tailed unpaired t test was performed to determine the statistical significance.

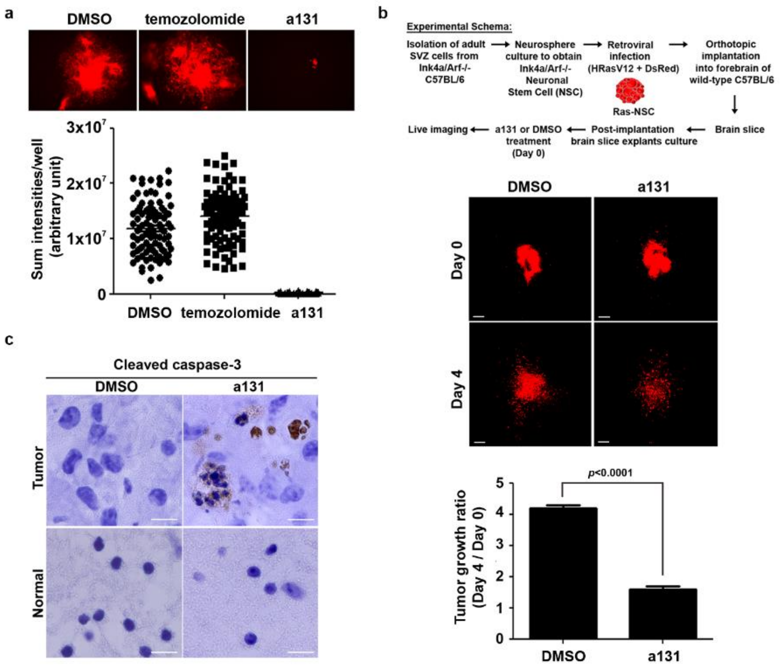
a
DMSO
temozolomide
a131
Sum intensities/well (arbitrary unit)
3x10^7
2x10^7
1x10^7
0
DMSO temozolomide a131
b
Experimental Schema:
Isolation of adult SVZ cells from Ink4a/Arf-/- C57BL/6
Neurosphere culture to obtain Ink4a/Arf-/- Neuronal Stem Cell (NSC)
Retroviral infection (HRasV12 + DsRed)
Ras-NSC
Orthotopic implantation into forebrain of wild-type C57BL/6
Brain slice
Post-implantation brain slice explants culture
a131 or DMSO treatment (Day 0)
Live imaging
DMSO
a131
Day 0
Day 4
p<0.0001
Tumor growth ratio (Day 4 / Day 0)
DMSO
a131
c
Cleaved caspase-3
DMSO
a131
Tumor
Normal

Supplementary Figure 3 | a131 suppresses growth of Ras-driven glioma initiating cells (GIC).

(**a**) Effect of a131 on murine GICs sphere growth. Representative images of DsRed-expressing GICs incubated for 7 days in neural stem cell medium containing DMSO, temozolomide (100 μM) or a131(5 μM).

(**b**) Effect of a131 on tumor growth in murine brain explants. Schematic description of the experiment (top). Coronal slices established from the brains of tumor-bearing C57BL/6 mice before (Day 0) and after (Day 4) treatment with DMSO or a131 (20 μM). Red: DsRed-expressing GICs with scale bar: 300 μm (middle). SVZ: subventricular zone. Tumor growth ratio (Day 4/Day 0) was quantified and mean values with ± S.D. are shown from triplicated experiments (bottom). Two-tailed unpaired t test was performed to determine the statistical significance.

(**c**) Effect of a131 on tumor viability in murine brain explants. Immunostaining of cleaved-caspase-3 in coronal brain slices treated with DMSO or a131 (20 μM) for 4 days. Scale bar: 20 μm.

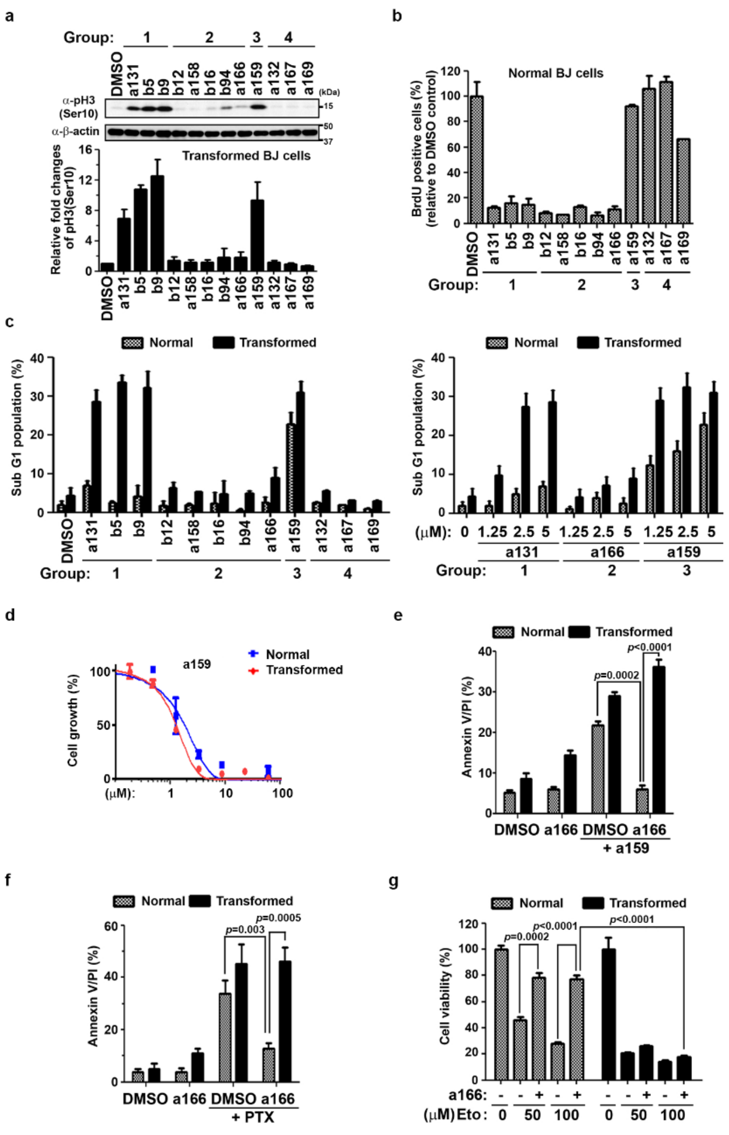
a
Group: 1 2 3 4
DMSO a131 b5 b9 b12 a158 b16 b94 a166 a159 a132 a167 a169
α-pH3 (Ser10)
α-β-actin
(kDa) 15 50 37
Transformed BJ cells
Relative fold changes of pH3(Ser10)
b
Normal BJ cells
BrdU positive cells (%) (relative to DMSO control)
c
Normal Transformed
Sub G1 population (%)
(μM): 0 1.25 2.5 5 1.25 2.5 5 1.25 2.5 5
a131 a166 a159
d
a159
Normal
Transformed
Cell growth (%)
(μM): 1 10 100
e
Annexin V/PI (%)
p=0.0002
p<0.0001
DMSO a166 DMSO a166
+ a159
f
Annexin V/PI (%)
p=0.003
p=0.0005
DMSO a166 DMSO a166
+ PTX
g
Cell viability (%)
p=0.0002
p<0.0001
p<0.0001
a166:
(μM) Eto: 0 50 100 0 50 100

Supplementary Figure 4 | Dual inhibitory properties of a131 responsible for its cancer-selective killing efficacy.

(**a-c**) Normal and transformed BJ cells were treated with a131 and its derivatives at 5 μM for 48 h.

(**a**) Immunoblot analysis to determine the ability of 131 and its derivatives to induce mitotic arrest in transformed BJ cells using phospho-histone H3(Ser10) [pH3(Ser10)] and β-actin (loading control) antibodies. The fold induction of band intensities [pH3(Ser10)/ β-actin] as compared to DMSO is plotted with mean values and ± S.D. (n=3).

(**b**) BrdU incorporation assay using normal BJ cells as in Fig. 1c. The percentage of cells with BrdU positive population in comparison with DMSO control is shown with mean values and ± S.D. (n=3).

(**c**) FACS analysis to determine the percentage of cells in subG1 (<2N). Mean values and ± S.D. (n=3) are shown. Indicated cells were treated with a131 and its derivatives at 5 μM (left) or at the indicated concentration (right). Note that only Group 1 compounds retain the ability to selectively kill transformed BJ cells, while compounds in Group 3 killed both normal and transformed cell lines with much less selectivity than those in Group 1.

(**d**) Normal and transformed BJ cells were treated with a159 at a range of different concentrations for 72 h in triplicate and cell viability was determined by MTT assay. Mean values with ± standard deviation (S.D.) are shown (n=3).

(**e-g**) Normal and transformed BJ cells were treated with a166 at 5 μM. 48 h after treatment, cells were further treated with a159 (**e**), paclitaxel (PTX) (**f**) or etoposide (**g**) for additional 48-72 h.

(**e, f**) FACS analysis of cells stained with Annexin V together with PI. The percentage of double positive cells for Annexin V and PI is shown with mean values and ± S.D. (n=3).

(**g**) MTT assay for cell viability. The results are plotted in comparison with DMSO control with mean values and ± S.D. (n=4).

Where indicated, two-tailed unpaired t test was performed to determine the statistical significance.

a

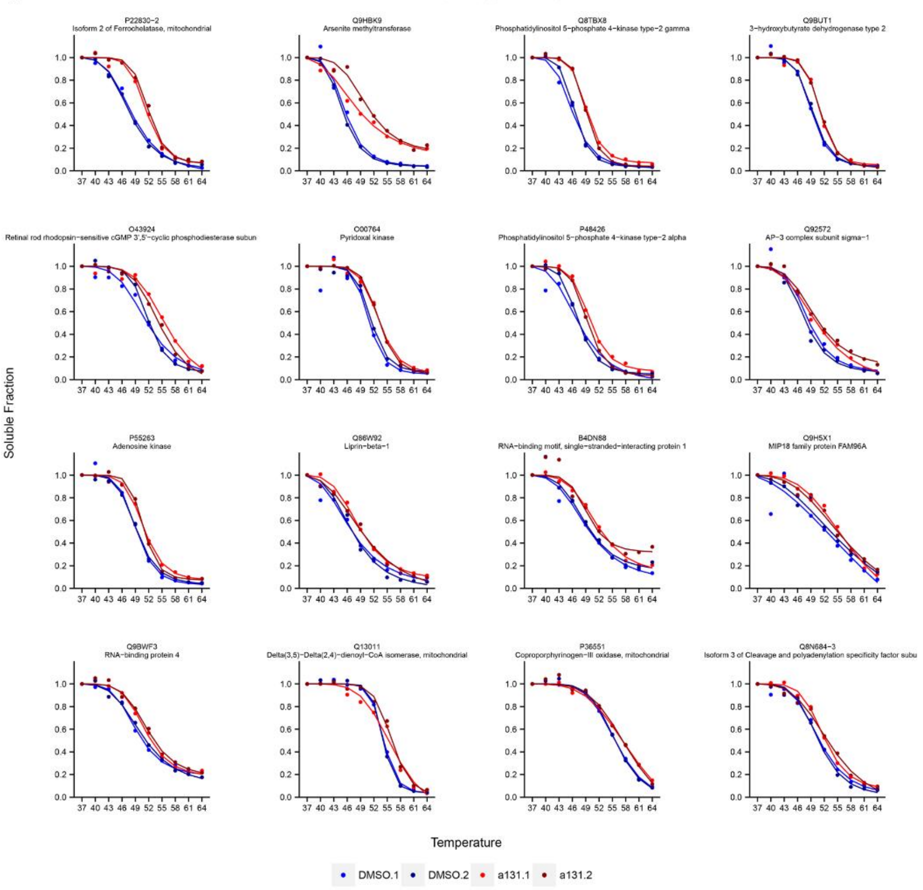

CETSA data plotting_curve fitting
P22830-2
Isoform 2 of Ferrochelatase, mitochondrial
Q9HBK9
Arsenite methyltransferase
Q8TBX8
Phosphatidylinositol 5-phosphate 4-kinase type-2 gamma
Q9BUT1
3-hydroxybutyrate dehydrogenase type 2
O43924
Retinal rod rhodopsin-sensitive cGMP 3',5'-cyclic phosphodiesterase subun
O00764
Pyridoxal kinase
P48426
Phosphatidylinositol 5-phosphate 4-kinase type-2 alpha
Q92572
AP-3 complex subunit sigma-1
P55263
Adenosine kinase
Q86W92
Liprin-beta-1
B4DN88
RNA-binding motif, single-stranded-interacting protein 1
Q9H5X1
MIP18 family protein FAM96A
Q9BWF3
RNA-binding protein 4
Q13011
Delta(3,5)-Delta(2,4)-dienoyl-CoA isomerase, mitochondrial
P36551
Coproporphyrinogen-III oxidase, mitochondrial
Q8N684-3
Isoform 3 of Cleavage and polyadenylation specificity factor subu
Soluble Fraction
0.0 0.2 0.4 0.6 0.8 1.0
37 40 43 46 49 52 55 58 61 64
Temperature
DMSO.1
DMSO.2
a131.1
a131.2

b

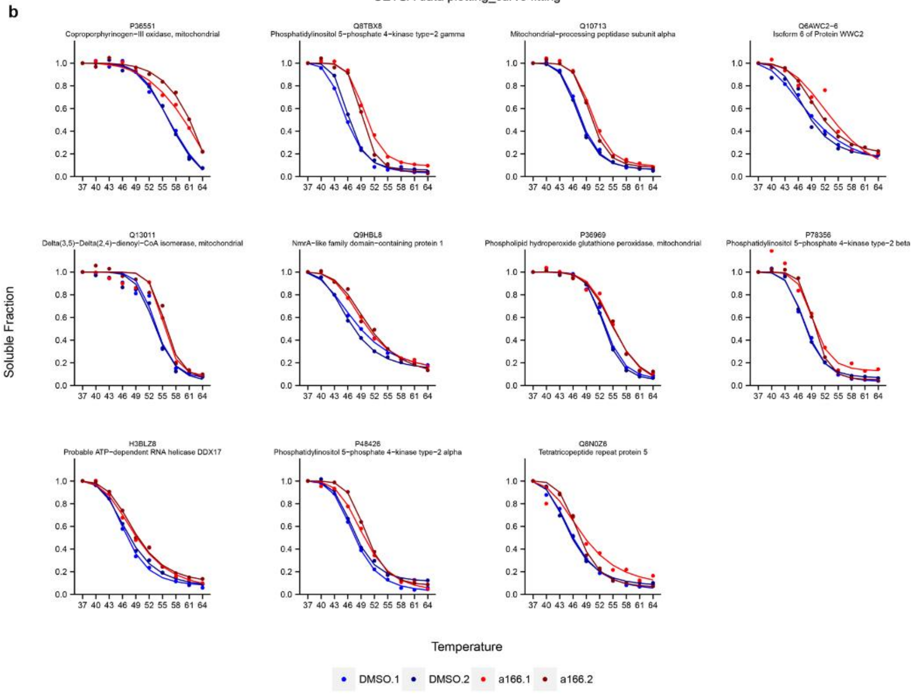

CETSA data plotting_curve fitting
P36551
Coproporphyrinogen-III oxidase, mitochondrial
Q8TBX8
Phosphatidylinositol 5-phosphate 4-kinase type-2 gamma
Q10713
Mitochondrial-processing peptidase subunit alpha
Q6AWC2-6
Isoform 6 of Protein WWC2
Q13011
Delta(3,5)-Delta(2,4)-dienoyl-CoA isomerase, mitochondrial
Q9HBL8
NmrA-like family domain-containing protein 1
P36969
Phospholipid hydroperoxide glutathione peroxidase, mitochondrial
P78356
Phosphatidylinositol 5-phosphate 4-kinase type-2 beta
H3BLZ8
Probable ATP-dependent RNA helicase DDX17
P48426
Phosphatidylinositol 5-phosphate 4-kinase type-2 alpha
Q8N0Z6
Tetratricopeptide repeat protein 5
Soluble Fraction
0.0 0.2 0.4 0.6 0.8 1.0
37 40 43 46 49 52 55 58 61 64
Temperature
DMSO.1
DMSO.2
a166.1
a166.2

Supplementary Figure 5 | CETSA melt curves for prominent hits with a131 and a166 in normal BJ cell lysates.

(**a**) Normal BJ cell lysates were treated with vehicle control (DMSO) or a131 compound and then subjected to CETSA treatment. CETSA melt curves for the 16 protein hits that passed the selection criteria are shown. Curves marked in blue represent the DMSO control treated samples and in red show the a131 treated cell lysates.

(**b**) Normal BJ cell lysates were treated with vehicle control (DMSO) or a166 compound and then subjected to CETSA treatment. CETSA melt curves for the 11 protein hits that passed the selection criteria are shown. Curves marked in blue represent the DMSO control treated samples and in red show the a166 treated cell lysates.

Data is presented as two individual replicates for each condition from one representative experiment.

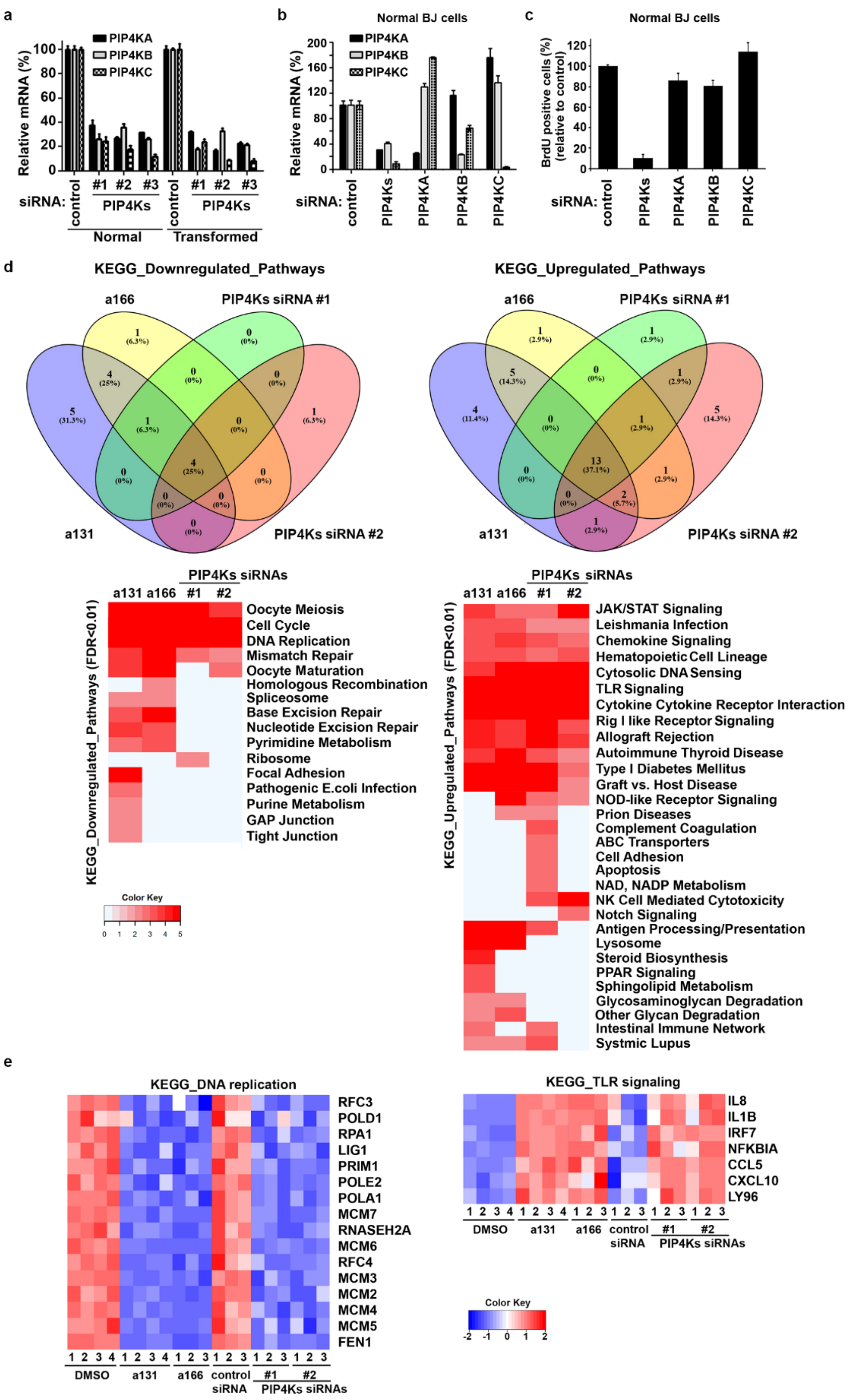

a
Relative mRNA (%)
PIP4KA
PIP4KB
PIP4KC
siRNA:
control
#1 #2 #3
PIP4Ks
Normal
Transformed
b
Normal BJ cells
c
BrdU positive cells (%) (relative to control)
d
KEGG_Downregulated_Pathways
KEGG_Upregulated_Pathways
a166
PIP4Ks siRNA #1
a131
PIP4Ks siRNA #2
PIP4Ks siRNAs
KEGG_Downregulated_Pathways (FDR<0.01)
Oocyte Meiosis
Cell Cycle
DNA Replication
Mismatch Repair
Oocyte Maturation
Homologous Recombination
Spliceosome
Base Excision Repair
Nucleotide Excision Repair
Pyrimidine Metabolism
Ribosome
Focal Adhesion
Pathogenic E.coli Infection
Purine Metabolism
GAP Junction
Tight Junction
Color Key
KEGG_Upregulated_Pathways (FDR<0.01)
JAK/STAT Signaling
Leishmania Infection
Chemokine Signaling
Hematopoietic Cell Lineage
Cytosolic DNA Sensing
TLR Signaling
Cytokine Cytokine Receptor Interaction
Rig I like Receptor Signaling
Allograft Rejection
Autoimmune Thyroid Disease
Type I Diabetes Mellitus
Graft vs. Host Disease
NOD-like Receptor Signaling
Prion Diseases
Complement Coagulation
ABC Transporters
Cell Adhesion
Apoptosis
NAD, NADP Metabolism
NK Cell Mediated Cytotoxicity
Notch Signaling
Antigen Processing/Presentation
Lysosome
Steroid Biosynthesis
PPAR Signaling
Sphingolipid Metabolism
Glycosaminoglycan Degradation
Other Glycan Degradation
Intestinal Immune Network
Systmic Lupus
e
KEGG_DNA replication
RFC3
POLD1
RPA1
LIG1
PRIM1
POLE2
POLA1
MCM7
RNASEH2A
MCM6
RFC4
MCM3
MCM2
MCM4
MCM5
FEN1
DMSO
a131
a166
control siRNA
#1
#2
PIP4Ks siRNAs
KEGG_TLR signaling
IL8
IL1B
IRF7
NFKBIA
CCL5
CXCL10
LY96

Supplementary Figure 6 | Gene expression similarity between a131 and a166 treatment and PIP4Ks knockdown with phenocopy of growth arrest in normal BJ cells.

(**a, b**) Quantitative real-time PCR (qRT-PCR) analysis to measure mRNA abundance of individual PIP4K family member in triplicated experiments. Normal and transformed BJ cells were transfected with three different sets of siRNAs to target all PIP4Ks (**a**) or indicated individual siRNA to target individual PIP4K isoform (**b**) as described in the materials and methods.

(**c**) BrdU incorporation assay as in Fig. 1c. Normal BJ cells were transfected with either control non-silencing or indicated individual or combined PIP4Ks siRNAs for 48h. The percentage of cells with BrdU positive population in comparison with control non-silencing siRNA is shown with mean values and ± S.D. (n=3).

(**d**) Gene expression data from normal BJ cells treated with a131 and a166 for 24h or transfected with two different sets of PIP4Ks for 48h were used to identify enriched KEGG pathways. Unique and overlapping pathways were identified through separate 4-way Venn diagrams (top) and heatmaps (bottom) of up- and down-regulated pathway lists, respectively. Pathways that were significant at FDR<1% were selected from each of the 4 studies and compared via the Venny software package (http://bioinforgp.cnb.csic.es/tools/venny/index.html).

(**e**) There are a total of 4 down-regulated and 12 up-regulated KEGG pathways at FDR<0.01 across all 4 experiments. In addition to cell cycle pathway as in Fig. 2F, genes contributing to core enrichment of the selected 2 pathways (DNA replication, Toll-like receptor signaling) are shown as examples of the effect for down-regulated and up-regulated pathways. The per-sample expression profiles of these genes are depicted in the heatmap using an intensity-based, row-normalized color scale from blue to red, with blue indicating lower expression.

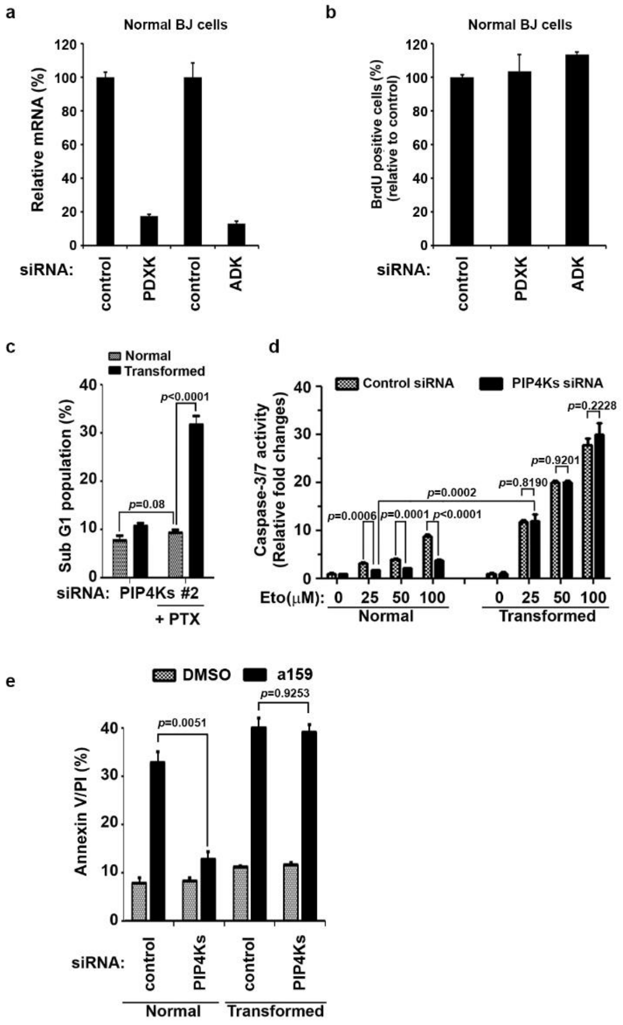
a
Normal BJ cells
Relative mRNA (%)
siRNA:
control
PDXK
control
ADK
b
Normal BJ cells
BrdU positive cells (%) (relative to control)
siRNA:
control
PDXK
ADK
c
Normal
Transformed
Sub G1 population (%)
p=0.08
p<0.0001
siRNA: PIP4Ks #2
+ PTX
d
Control siRNA
PIP4Ks siRNA
Caspase-3/7 activity (Relative fold changes)
p=0.0006
p=0.0001
p<0.0001
p=0.0002
p=0.8190
p=0.9201
p=0.2228
Eto(μM): 0 25 50 100 0 25 50 100
Normal
Transformed
e
DMSO
a159
Annexin V/PI (%)
p=0.0051
p=0.9253
siRNA:
control
PIP4Ks
control
PIP4Ks
Normal
Transformed

Supplementary Figure 7 | PIP4Ks knockdown with phenocopy of the chemoprotective effects of a166.

(**a**) qRT-PCR analysis to measure mRNA abundance of PDXK and ADK in triplicated experiments. Normal BJ cells were transfected with indicated siRNAs to target PDXK and ADK.

(**b**) BrdU incorporation assay as in Fig. 1c. Normal BJ cells were transfected with either control non-silencing or indicated siRNAs for 48h. The percentage of cells with BrdU positive population in comparison with control non-silencing siRNA is shown with mean values and ± S.D. (n=3).

(**c-e**) Normal and transformed BJ cells were transfected with either control non-silencing or PIP4Ks siRNAs for 48h and subsequently treated with paclitaxel (PTX) (**d**), etoposide (**e**) or a159 for additional 48-72 h.

(**c**) FACS analysis with PI staining. The percentage of cells in subG1 (<2N) is shown with mean values and ± S.D. (n=3).

(**d**) Caspsase-3/7 activity assay for apoptosis. The fold induction in comparison with DMSO control is plotted with mean values and ± S.D. (n=4).

e) FACS analysis of cells stained with Annexin V together with PI. The percentage of double positive cells for Annexin V and PI is shown with mean values and ± S.D. (n=3).

Where indicated, two-tailed unpaired t test was performed to determine the statistical significance.

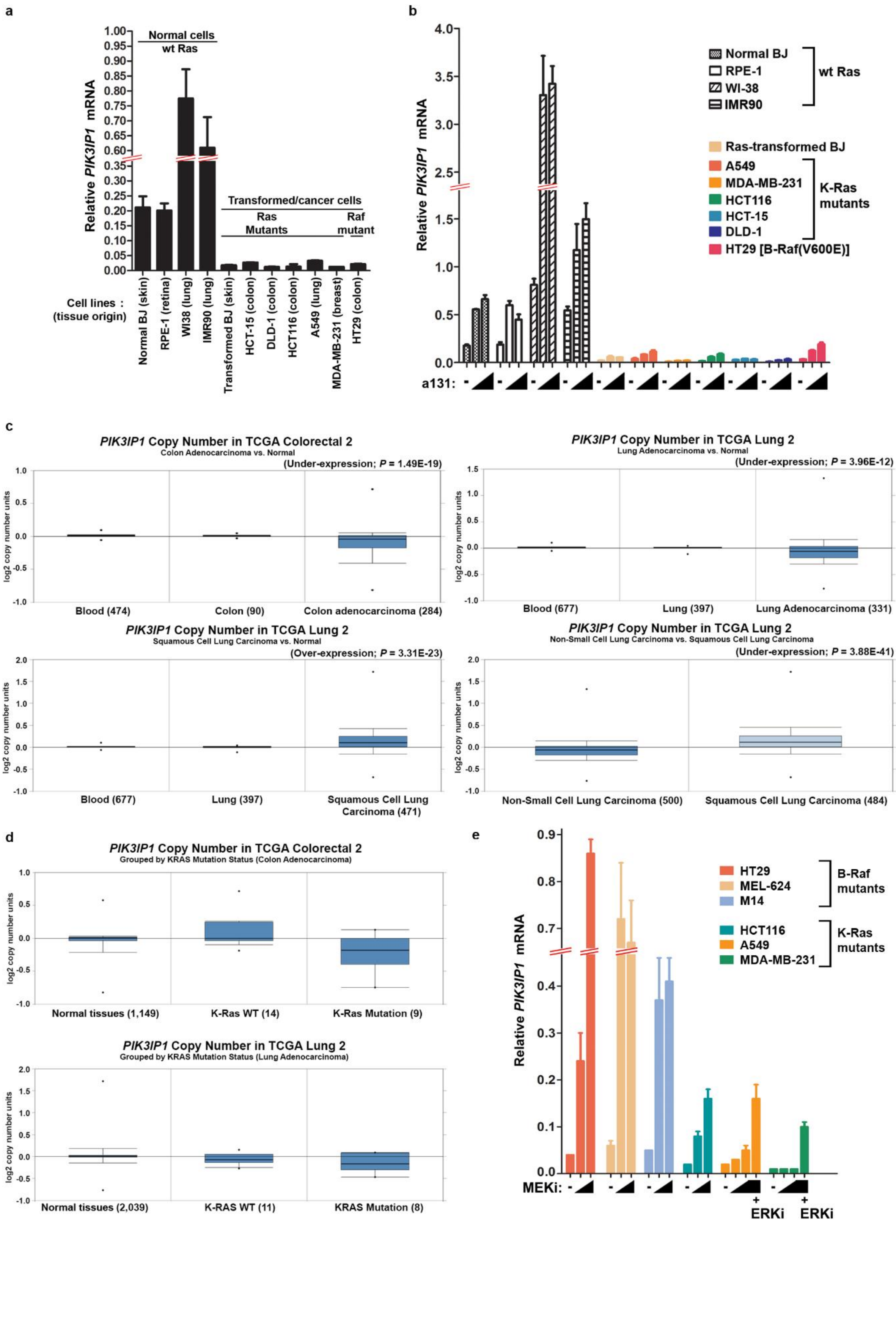
a
Normal cells
wt Ras
Transformed/cancer cells
Ras Mutants
Raf mutant
Relative PIK3IP1 mRNA
Cell lines :
(tissue origin)
Normal BJ (skin)
RPE-1 (retina)
WI38 (lung)
IMR90 (lung)
Transformed BJ (skin)
HCT-15 (colon)
DLD-1 (colon)
HCT116 (colon)
A549 (lung)
MDA-MB-231 (breast)
HT29 (colon)
b
Relative PIK3IP1 mRNA
Normal BJ
RPE-1
WI-38
IMR90
wt Ras
Ras-transformed BJ
A549
MDA-MB-231
HCT116
HCT-15
DLD-1
K-Ras mutants
HT29 [B-Raf(V600E)]
a131:
c
PIK3IP1 Copy Number in TCGA Colorectal 2
Colon Adenocarcinoma vs. Normal
(Under-expression; P = 1.49E-19)
log2 copy number units
Blood (474)
Colon (90)
Colon adenocarcinoma (284)
PIK3IP1 Copy Number in TCGA Lung 2
Lung Adenocarcinoma vs. Normal
(Under-expression; P = 3.96E-12)
Blood (677)
Lung (397)
Lung Adenocarcinoma (331)
PIK3IP1 Copy Number in TCGA Lung 2
Squamous Cell Lung Carcinoma vs. Normal
(Over-expression; P = 3.31E-23)
Blood (677)
Lung (397)
Squamous Cell Lung Carcinoma (471)
PIK3IP1 Copy Number in TCGA Lung 2
Non-Small Cell Lung Carcinoma vs. Squamous Cell Lung Carcinoma
(Under-expression; P = 3.88E-41)
Non-Small Cell Lung Carcinoma (500)
Squamous Cell Lung Carcinoma (484)
d
PIK3IP1 Copy Number in TCGA Colorectal 2
Grouped by KRAS Mutation Status (Colon Adenocarcinoma)
Normal tissues (1,149)
K-Ras WT (14)
K-Ras Mutation (9)
PIK3IP1 Copy Number in TCGA Lung 2
Grouped by KRAS Mutation Status (Lung Adenocarcinoma)
Normal tissues (2,039)
K-RAS WT (11)
KRAS Mutation (8)
e
Relative PIK3IP1 mRNA
HT29
MEL-624
M14
B-Raf mutants
HCT116
A549
MDA-MB-231
K-Ras mutants
MEKi:
+
ERKi
+
ERKi

Supplementary Figure 8 | *PIK3IP1* mRNA expression in various normal and Ras- or Raf-mutant cancer cell lines and across indicated cancer-normal and cancer-cancer using TCGA dataset.

(**a, b, e**) qRT-PCR analysis of *PIK3IP1* mRNA expression in various normal and Ras- or Raf-mutant cancer cell lines.

(**a**) Endogenous *PIK3IP1* mRNA expression levels.

(**b**) Indicated cells were treated with DMSO control or a131 at 2.5 and 5 μM for 24 h.

(**c, d**) Oncomine analysis of PIK3IP gene expression.

(**c**) *PIK3IP1* mRNA expression is suppressed in human colorectal and lung adenocarcinomas where Ras mutations and activation of Ras signaling pathways are common compared with their corresponding normal tissues or squamous cell lung carcinoma where Ras mutations are uncommon. Expression microarray results of the TCGA consortium data set were analyzed, and statistical significance was calculated using the Oncomine website (www.oncomine.org).

(**d**) Negative correlations between *PIK3IP1* mRNA expression and Ras mutation status in human colorectal and lung adenocarcinomas.

Box plots show differences in mRNA expression across indicated cancer-normal and cancer-cancer. Data are presented as box plot distribution (line = median value). Numbers represent samples analyzed.

(**e**) Pharmacological inhibition of MEK/ERK attenuates Ras- and Raf-mediated suppression of PIK3IP1. Various Ras- or Raf-mutant cancer cell lines were treated with MEK inhibitor (MEKi) U0126 and/or ERK inhibitor (ERKi) SCH772984 for 24h and *PIK3IP1* mRNA expression was measured by qRT-PCR. Of note, increase in *PIK3IP1* mRNA expression was much prominent in Raf-mutant cancer cells, suggesting the high MAPK activity is responsible for the suppression of *PIK3IP1*.

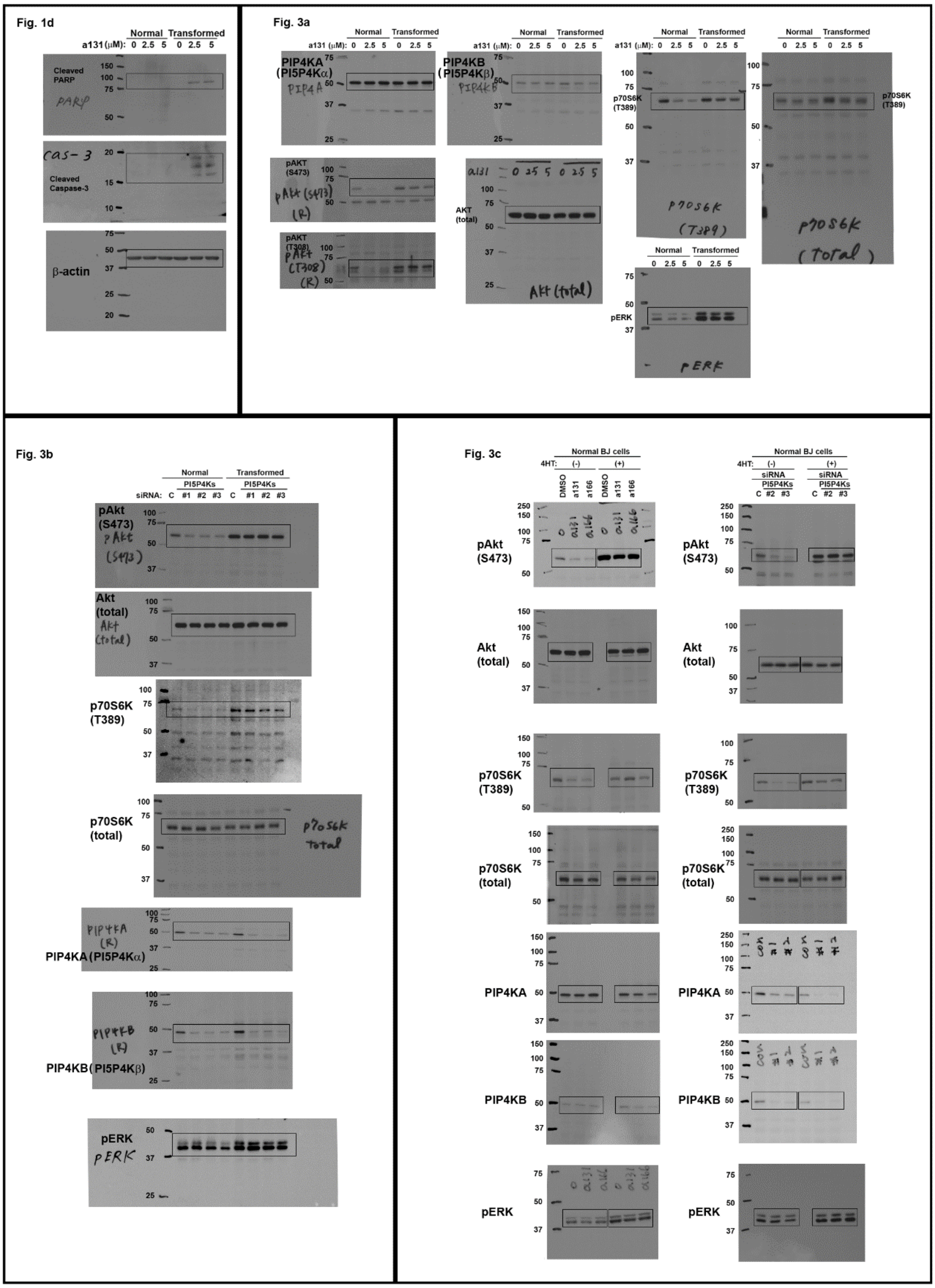
Fig. 1d
Normal Transformed
a131 (μM): 0 2.5 5 0 2.5 5
Cleaved PARP
Cleaved Caspase-3
β-actin
Fig. 3a
PIP4KA (PI5P4Kα)
PIP4KB (PI5P4Kβ)
pAKT (S473)
pAKT (T308)
AKT (total)
p70S6K (T389)
p70S6K
pERK
Fig. 3b
Normal Transformed
PI5P4Ks PI5P4Ks
siRNA: C #1 #2 #3 C #1 #2 #3
pAkt (S473)
Akt (total)
p70S6K (T389)
p70S6K (total)
PIP4KA (PI5P4Kα)
PIP4KB (PI5P4Kβ)
pERK
Fig. 3c
Normal BJ cells
4HT: (-) (+)
DMSO a131 a166 DMSO a131 a166
siRNA PI5P4Ks
C #2 #3 C #2 #3
pAkt (S473)
Akt (total)
p70S6K (T389)
p70S6K (total)
PIP4KA
PIP4KB
pERK

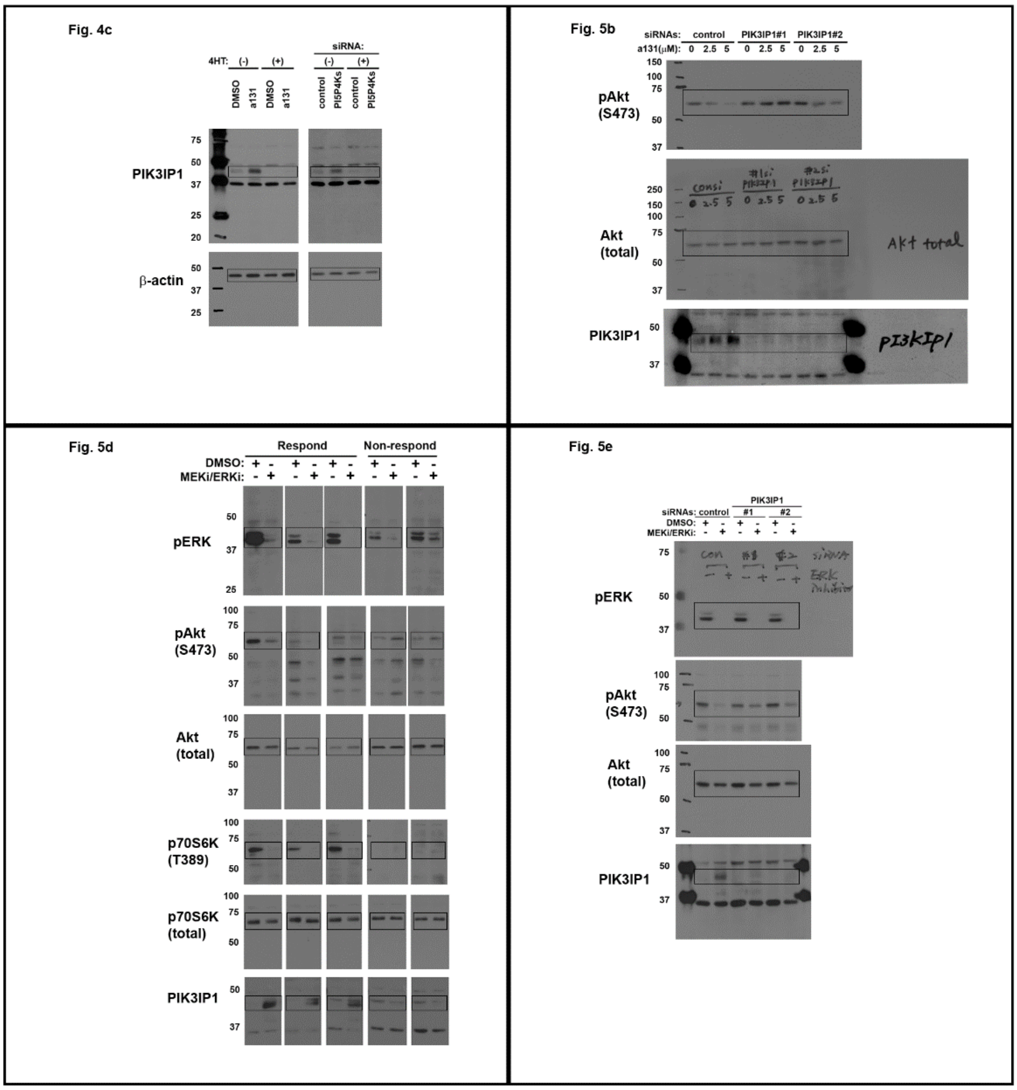

Supplementary Figure 9 | Uncut blots. The black box regions are presented in the indicated figures in the manuscript.
