# Supplementary material for: Dual blockade of the lipid kinase PIP4Ks and mitotic pathways leads to cancer-selective lethality
Source: Nat Commun. 2017 Dec 19;8:2200. doi: 10.1038/s41467-017-02287-5 (PMC5736559; doi:10.1038/s41467-017-02287-5)
Supplement: Supplementary file 3 — Description of Additional Supplementary Files [file 41467_2017_2287_MOESM3_ESM.pdf]

Description of Additional Supplementary Files

File Name: Supplementary Movie 1
Description: Mitotic progression of Ras-transformed BJ cells treated with DMSO.

File Name: Supplementary Movie 2
Description: Mitotic progression of Ras-transformed BJ cells treated with a131.

File Name: Supplementary Movie 3
Description: Mitotic progression of normal BJ cells treated with DMSO.

File Name: Supplementary Movie 4
Description: Mitotic progression of normal BJ cells treated with a131.

File Name: Supplementary Data 1
Description: List of normal and cancer cell lines and culture media used in this study.

File Name: Supplementary Data 2
Description: List of compounds with their structures clarified into 4 groups based on a131 SAR analysis.

File Name: Supplementary Data 3
Description: List of CETSA hits that passed the selection criteria for a131 and a166 datasets.
